# Modulation of early auditory processing by visual information: Prediction or bimodal integration?

**DOI:** 10.3758/s13414-021-02240-1

**Published:** 2021-01-27

**Authors:** Maria V. Stuckenberg, Erich Schröger, Andreas Widmann

**Affiliations:** 1 Institute of Psychology, Leipzig University, Neumarkt 9-19, D-04109 Leipzig, Germany; 2 Max Planck Institute for Human Cognitive and Brain Sciences, Stephanstraße 1A, D-04103 Leipzig, Germany; 3 Leibniz Institute for Neurobiology, Brenneckestraße 6, D-39118 Magdeburg, Germany

**Keywords:** Audition, Methods: EEG, MEG, Multisensory processing

## Abstract

What happens if a visual cue misleads auditory expectations? Previous studies revealed an early visuo–auditory incongruency effect, so-called incongruency response (IR) of the auditory event-related brain potential (ERP), occurring 100 ms after onset of the sound being incongruent to the preceding visual cue. So far, this effect has been ascribed to reflect the mismatch between auditory sensory expectation activated by visual predictive information and the actual sensory input. Thus, an IR should be confined to an asynchronous presentation of visual cue and sound. Alternatively, one could argue that frequently presented congruent visual-cue–sound combinations are integrated into a bimodal representation whereby violation of the visual–auditory relationship results in a bimodal feature mismatch (the IR should be obtained with asynchronous and with synchronous presentation). In an asynchronous condition, an either high-pitched or low-pitched sound was preceded by a visual note symbol presented above or below a fixation cross (90% congruent; 10% incongruent), while in a synchronous condition, both were presented simultaneously. High-pitched and low-pitched sounds were presented with different probabilities (83% vs. 17%) to form a strong association between bimodal stimuli. In both conditions, tones with pitch incongruent with the location of the note symbols elicited incongruency effects in the N2 and P3 ERPs; however, the IR was only elicited in the asynchronous condition. This finding supports the sensorial prediction error hypothesis stating that the amplitude of the auditory ERP 100 ms after sound onset is enhanced in response to unexpected compared with expected but otherwise identical sounds.

**Supplementary Information:**

The online version contains supplementary material available at 10.3758/s13414-021-02240-1.

The benefits of visual information on the processing of auditory information have been observed in several studies—more precisely, a variety of studies looked at the processing of simultaneously presented visual and auditory stimuli. If both stimuli occurred concurrently and are task relevant, the processing of the stimuli was facilitated as indicated by reduced reaction times, improved accuracies, and effects in event-related potentials (ERPs) with bimodal compared with auditory-only stimulation (e.g., Giard & Peronnet, 1999). But most studies do not find differences in early auditory processing (i.e., in the N1 range) between bimodal and auditory-only presentations (speech: Miki, Watanabe, & Kakigi, 2004; nonspeech: Fort, Delpuech, Pernier, & Giard, 2002; Giard & Peronnet, 1999). Nevertheless, in a natural environment, the visual information that we get from lip movements is generally present before the speech sound (on average 150 ms earlier; Chandrasekaran, Trubanova, Stillittano, Caplier, & Ghazanfar, 2009). Therefore, several authors investigated the effect of anticipatory visual information on auditory processing and revealed that auditory evoked potentials are attenuated and speeded up during audiovisual compared with auditory-only processing (Stekelenburg & Vroomen, 2007; Vroomen & Stekelenburg, 2010). These studies report N1 modulation (i.e., N1 suppression) for bimodal versus unimodal stimulation. This attenuation of the auditory N1 has been explained by auditory prediction generated by the preceding visual information.

Widmann, Kujala, Tervaniemi, Kujala, and Schröger (2004) investigated the role of visually based predictions on auditory sensory processing in a symbol-to-sound mapping paradigm. They presented a sequence of score-like visual symbols prior to a corresponding auditory sequence. The sounds of the sequence were either all congruent with the preceding visual information or one sound of the sequence was incongruent with the visual score. The auditory ERPs were enhanced with incongruent compared with congruent (but otherwise identical) sounds about 100–130 ms after sound onset at frontolateral sites. It is assumed that this amplitude difference (i.e., negative deflection in the difference wave incongruent-minus-congruent) results from a mismatch between predicted and actually experienced stimuli. The predictive coding framework (Friston, 2005) claims that, based on the recent stimulation history, extrapolations of upcoming events can be made. If the current sensory input violates the prediction, a so-called prediction error is generated (for a review, see Bendixen, SanMiguel, & Schröger, 2012). In this context, the amplitude difference (incongruent-minus-congruent) was interpreted as an enhanced prediction error signal in response to incongruent (violating visual-based prediction) compared with congruent sounds and was termed “incongruency response” (IR). This implies that the preceding visual information was used to establish a certain expectation of the upcoming sound (Widmann et al., 2004). Elicitation of the IR is thought to not only mark a prediction error but more specifically a sensory prediction error since previous studies revealed auditory generators in the superior temporal gyrus (Pieszek, Widmann, Gruber, & Schröger, 2013). Hence, the presence of the IR reflects a sensorial prediction error between the established prediction of an auditory stimulus and the actually experienced sound. Meanwhile, the IR has been replicated in several studies (Pieszek, Schröger, & Widmann, 2014; Pieszek et al., 2013; Stuckenberg, Schröger, & Widmann, 2019).

Another explanation for the observed IR besides sensorial prediction error could be bimodal feature mismatch between quickly extracted auditory and visual information in a bimodal representation. It has been shown that the auditory sensory memory is sensitive to feature binding. This was mainly done by investigating the modulation of the so-called mismatch negativity (MMN). The MMN is the negative deflection apparent in the ERP difference wave of deviant (rarely occurring) and repeatedly presented standard sounds, usually 100–200 ms after sound onset with a maximum over frontal and central scalp locations. It was first described by Näätänen and colleagues (Näätänen, Gaillard, & Mäntysalo, 1978) and is thought to represent auditory stimulus representation. In general, conjunctions between two sound features can be established by a frequent presentation of a particular feature relationship. Whereby a violation of this relationship (a change of one sound feature while the other one remains unaltered) results in an MMN. Feature conjunctions have been observed using frequency–intensity (Gomes, Bernstein, Ritter, Vaughan, & Miller, 1997; Paavilainen, Simola, Jaramillo, Näätänen, & Winkler, 2001; Takegata, Paavilainen, Näätänen, & Winkler, 1999), timbre–pitch (Takegata et al., 2005), and frequency–location relationships (Sussman, Gomes, Nousak, Ritter, & Vaughan, 1998; Winkler, Czigler, Sussman, Horváth, & Balázs, 2005). Furthermore, the visual equivalent, the visual MMN, has been observed when frequently presented grating orientation–color relationships were violated (Winkler et al., 2005). In a bimodal paradigm, we can imagine the following scenario. One of two possible visual stimuli is frequently presented together with one of two possible auditory stimuli. Thereby, two types of frequently presented congruent feature conjunctions are possible (e.g., high visual cue and high sound or low visual cue and low sound) whereby the incongruent feature conjunctions are presented less frequently (e.g., high visual cue and low sound or low visual cue and high sound). If visual and auditory stimuli are presented with close temporal proximity, they can be perceived as a unified bimodal representation that consists of two frequently presented congruent conjunctions. The two rare incongruent conjunctions most likely would elicit a mismatch response. For instance, Ullsperger, Erdmann, Freude, and Dehoff (2006) have shown that auditory mismatch responses can be induced by visual changes (whereby auditory information keeps constant). They presented a picture of a hammer hitting a nail simultaneously with a “bang” sound as the standard visual and auditory presentation (79% of trials). In addition, a deviant picture with the hammer hitting the finger and a deviant “ouch” sound were rarely presented (9.9% of trials). More interestingly, they introduced a condition where the deviant picture was presented together with the standard sound (9.9% of trials). They found an MMN not only in classical comparison of deviant-minus-standard auditory processing, but also a standard sound presented with a deviant picture compared with a standard sound presented with a standard picture produced an enhanced negativity around 100 ms after sound onset. This shows that the regular presentation of a bimodal stimulation forms a certain bimodal percept. If this bimodal regularity is violated (here, by a differing visual stimulus), a bimodal error signal is elicited. In the context of our study, this would mean that the IR might reflect a mismatch of the unified bimodal percept and not necessarily of a visual-information-based sensorial prediction error.

Thus, the present study tested whether the IR either reflects sensorial prediction error or bimodal feature mismatch between quickly extracted auditory and visual information.

Several studies claim that the prediction effect induced by preceding visual information consists in N1 suppression (e.g., Stekelenburg & Vroomen, 2007; Vroomen & Stekelenburg, 2010). However, in these studies, ERPs elicited by bimodal versus unimodal presentations were contrasted. We conducted a study directly comparing the auditory processing of a condition where a visual-based prediction can be established (asynchronous presentation of visual and auditory stimuli) and a condition where no visual-based prediction can be established since the visual and auditory stimuli are presented simultaneously, but the context is optimal for bimodal integration (synchronous condition). As an advantage compared with previous studies, contrasting bimodal and unimodal processing, we presented two bimodal situations where the only difference relied in the predictability of the auditory stimulus.

Our experimental setup was adapted from studies by Pieszek et al. (2013) and Stuckenberg et al. (2019). We presented an eighth note symbol either above or below the fixation cross, which was followed (asynchronous condition) or accompanied (synchronous condition) by an either high-pitched or low-pitched sound. In 90% of the trials, visual and auditory stimuli were both high or both low (congruent). The incongruent combinations were presented less frequently (10% of the trials).

The main hypothesis was that if the IR would be elicited within the asynchronous but not within the synchronous condition, the IR would be validated as a prediction error signal and consequently would show that the auditory sensory memory was sensitive to the visual information. Based on several previous studies (Pieszek et al., 2013; Pieszek et al., 2014; Stuckenberg et al., 2019), we assume to observe an IR in the asynchronous condition confirming the visual-based sensorial prediction error hypothesis. If we observe an IR in the asynchronous and synchronous presentation condition, this would suggest that the IR rather reflects bimodal feature mismatch (i.e., mismatch of the unified bimodal percept).

## Materials and methods

### Participants

The electroencephalogram (EEG) and behavioral data of 20 participants were recorded. Due to an interrupted session, one data set had to be excluded. The remaining 19 participants (12 women; age range: 18–32 years; mean age = 21.8 years; 18 right-handed) reported normal hearing, normal or corrected-to-normal vision, and were not taking any medication affecting the central nervous system. All participants gave their written consent according to the Declaration of Helsinki and received either credit points or modest financial compensation for their participation. The project was approved by the local ethical committee of the Medical Faculty of the Leipzig University (AZ 089-16-14032016).

### Apparatus and stimuli

**Fig. 1:**
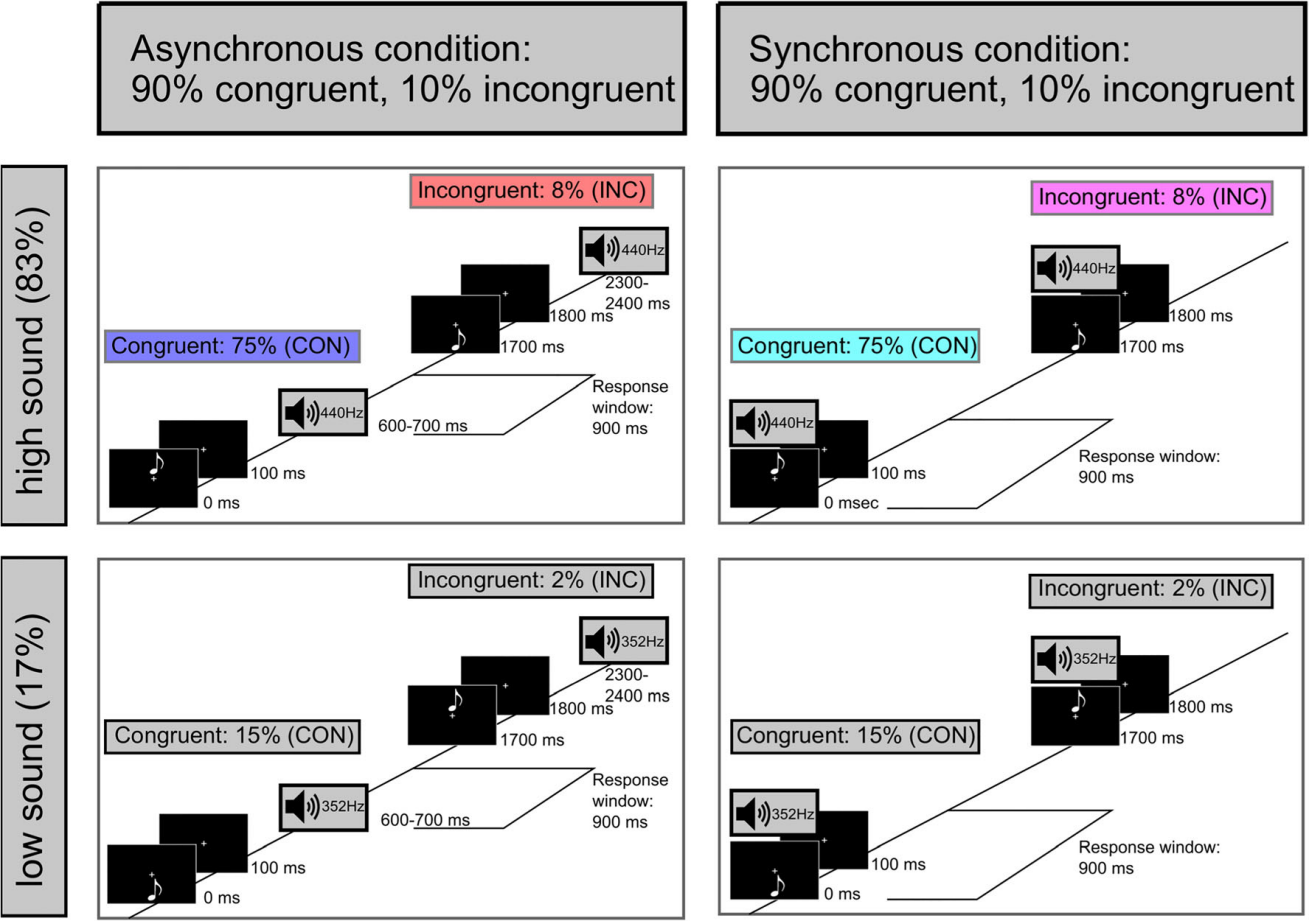
Schematic of the experimental design. Asynchronous (left) and synchronous (right) presentation conditions. In the asynchronous condition, the visual stimulus preceded the auditory stimulus by 600 ms. In the synchronous condition, visual and auditory stimuli were presented simultaneously. The response window started in both conditions with the presentation of the auditory stimulus and lasted 900 ms. The participants’ task was to indicate the pitch of the sound via button press. In both conditions, 90% of the trials were congruent (CON: visual and auditory stimulus either above fixation cross and high-pitched sound or below fixation cross and low-pitched sound) and 10% of the trials were incongruent (INC: visual and auditory stimulus either above fixation cross and low-pitched sound or below fixation cross and high-pitched sound). The probability of each visual-cue–sound combination is indicated. One of the tones (counterbalanced across participants) was presented more frequently (top row: standard tone, 83%) than the other tone (bottom row: deviant tone, 17%). This results in a more frequent presentation of the congruent visual-cue–sound combination with the frequent tone (asynchronous condition: blue; synchronous condition: cyan). The same color code is used for the presentation of the results. Only the frequent tone conditions (CON: blue/cyan and INC: red/magenta) are considered for analyses to investigate the elicitation of an IR. (Color figure online)

In the beginning of the experimental session, participants completed a general questionnaire (regarding personal information) and a shortened German version of the Edinburgh Handedness Inventory (Oldfield, 1971). During the experimental task, EEG was continuously recorded while participants were seated in an acoustically attenuated and electrically shielded chamber. The stimulation was presented with Psychophysics Toolbox (Version 3; Brainard, 1997; Kleiner et al., 2007) on a Linux-based system using GNU Octave (Version 4.0.0). The experimental setup was identical to the 83/17 sound probability condition in Stuckenberg et al. (2019). The paradigm consisted of an asynchronous and a synchronous condition, which were measured block-wise and were counterbalanced across participants. For half of the participants, the first half of the experiment consisted of the presentation of asynchronous blocks and the second half of synchronous blocks. For the other half of the participants, it was the other way around. Within the asynchronous condition (see Fig. 1, left), a white eighth note symbol (0.7° × 0.9° of visual angle) was presented either above or below the fixation cross (0.3° × 0.3° of visual angle) for 100 ms. This was followed, 600 ms after the visual stimulus onset, by the presentation of an either high-pitched (440 Hz) or low-pitched (352 Hz) sound for 100 ms. In other words, between the offset of the visual stimulus and the onset of the auditory stimulus the fixation cross was presented for 500 ms. Auditory stimuli were presented via loudspeakers (Bose Companion 2 Series II, Bose Corporation) that were installed left and right of the screen. Within the synchronous condition (see Fig. 1, right), the visual and the auditory stimulus were presented simultaneously for 100 ms. In both conditions, participants had to react to the pitch of the sound by pressing the corresponding button for high or low pitch (button associations—i.e., left vs. right button—were counterbalanced across participants). In addition, they were instructed to attend to the visual information serving as an indicator of the upcoming sound. A response window of 900 ms after sound onset was provided for the behavioral response in both conditions. The next trial started either 1,550, 1,700, or 1,850 ms (on average, 1,700 ms) after the onset of the previous visual stimulus.

We differentiated between congruent (CON) and incongruent (INC) visual-cue–sound combinations, whereby a congruent visual-cue–sound combination means that the visual symbol was presented above the fixation cross and the tone was high pitched, or that the visual symbol was presented below the fixation cross and the tone was low pitched. Congruent visual-cue–sound combinations were presented more frequently (90% of the trials) than incongruent (10% of the trials) visual-cue–sound combinations. In order to form a strong association between the visual and the auditory information, we used an increased presentation of one congruent visual-cue–sound combination. Stuckenberg et al. (2019) showed that this type of modulation (83/17 sound probability condition) facilitates the formation of a visual-based sensory prediction in a trial-by-trial experimental design. They only observed an IR elicitation when the probability of one tone was increased (83/17 condition) and not when both tones were presented with equal probability (50/50 condition). Therefore, in the present study, one of the tones (balanced across participants) was presented more frequently (in 83% of the trials) than the other tone (17% of the trials; i.e., standard vs. deviant tone). This led to an increased presentation probability of the corresponding congruent visual-cue–sound combination (as described in Stuckenberg et al., 2019). In 75% of the trials, the frequent tone is presented with a congruent visual stimulus (CON), whereby in 8% of the trials, the frequent tone is presented with an incongruent visual stimulus (INC). These two conditions were used in the present study because we were interested in the processing of misleading visual stimuli on early auditory processing. However, the use of a standard and a deviant tone resulted in two more conditions: when the deviant tone is presented with a congruent visual stimulus (15% of the trials) and when the deviant tone is presented with an incongruent visual stimulus (2% of the trials). The processing of the deviant tone was not analyzed in the present study since it reflects probability-based prediction processing and Pieszek et al. (2013) showed that these are distinct from the visual-based prediction processing of the standard tones that we were interested in. For each condition (asynchronous vs. synchronous), nine blocks were presented each containing 90 trials of the congruent condition (CON) and 10 trials of the incongruent condition (INC) presenting the standard tone. Furthermore, each block contained 18 congruent trials and two incongruent trials presenting the deviant tone. Each block lasted about 3.4 min. The trial order within each block was pseudorandomized. The first two trials of each block and at least one trial between two incongruent trials were always trials of the congruent condition (CON). Participants performed a training block before each blocked presentation condition (asynchronous vs. synchronous). The training block consisted of a shortened block (half the duration of a normal block) of the specific condition (either asynchronous or synchronous) and was used to familiarize the participants with the stimulation. During the whole experiment, occasional oral feedback regarding the participants’ performance was provided to keep them motivated. The experimental task itself had a duration of about 61.2 min.

### Data recording and analysis

The EEG was recorded from overall 64 active Ag/AgCl electrodes using a BrainAmp amplifier and the Vision Recorder 1.20 software (Brain Products). The active electrodes were placed on an EEG cap (actiCAP, Brain Products) according to the extended international 10–20 system (Chatrian, Lettich, & Nelson, 1985). In addition, electrodes were placed on the tip of the nose (reference electrode), on the center of the forehead (ground electrode) and on the left and right mastoid sites. In order to detect eye movements, two electrodes were placed on the outer canthi of the left and right eye, and one electrode was positioned below the left eye. The EEG was continuously recorded (500 Hz sampling rate) and amplified with 64 active electrodes and a BrainProducts ActiCap EEG system, using a nose-tip reference. For data analyses, the EEGLAB toolbox (Version 13; Delorme & Makeig, 2004) for MATLAB was used. Data preprocessing was identical to the methodology used in Stuckenberg et al. (2019). Data of both conditions (asynchronous and synchronous) were high-pass filtered with 0.1 Hz cutoff (finite impulse response [FIR] filter; Kaiser-windowed; Kaiser beta = 5.65; filter length = 9,056 points) and low-pass filtered with 48 Hz cutoff (finite impulse response [FIR] filter; Kaiser-windowed; Kaiser beta = 5.65; filter length = 1,812 points). Epochs were generated from −100 ms until +800 ms relative to the onset of the auditory stimulus. As described in Stuckenberg et al. (2019), we observed in the asynchronous condition contingent negative variation (CNV) potentials that were ongoing within a typical baseline window, since one visual stimulus was presented more frequently than the other one. The CNV potentials were more pronounced for the incongruent condition (i.e., the rarely presented visual stimulus). CNV potentials are typically observed when a task-relevant stimulus is regularly cued, reflecting response preparation (Walter, Cooper, Aldridge, McCallum, & Winter, 1964). It is common to filter the data with high cutoff values (2 Hz) to eliminate CNV potentials (e.g., Brown, Clarke, & Barry, 2007; Teder-Sälejärvi, McDonald, Di Russo, & Hillyard, 2002). The downside of this approach is that high cutoff values lead to an invalid interpretation of later ERP components (Luck, 2005; Widmann & Schröger, 2012; Widmann, Schröger, & Maess, 2015). To investigate which cutoff value suits best to eliminate potential CNV artifacts from the IR signal without altering the interpretation of later ERP components, we contrasted different high-pass filter cutoff values with each other (0.1 Hz, 0.5 Hz, 1.25 Hz and 1.5Hz). We did not observe main differences regarding IR elicitation (105–130 ms after sound onset; i.e., with all filter cutoff values we observed an IR elicitation in the asynchronous, but not in the synchronous condition. For a detailed comparison see Supplementary Material Fig. S1). We stuck to the 0.1 Hz high-pass filter cutoff since we were also interested in later ERP components. Nevertheless, to prevent contamination of the signal, we applied a baseline correction from 0 ms until +50 ms relative to onset of the auditory stimulus. In contrast to Stuckenberg et al. (2019), we used the same filter settings and baseline correction parameters for the asynchronous and the synchronous condition.

Further data preprocessing involved the identification of bad channels based on the deviation criterion (Bigdely-Shamlo, Mullen, Kothe, Su, & Robbins, 2015) that detects channels with unusually high-amplitude or low-amplitude deviation. The robust *z* score of the robust standard deviation is calculated for each channel. Values greater than 3 identify bad channels, which were removed from analysis and interpolated after the independent component analysis (ICA) was performed. An extended ICA was trained on 1-Hz filtered data (as recommended by Debener, Thorne, Schneider, & Viola, 2010) and nonoverlapping epochs with maximal length (−600 to 800 ms). The resulting ICA weights were saved on the 0.1 Hz high-pass filtered data. The faster and adjust criteria (Chaumon, Bishop, & Busch, 2015; Mognon, Jovicich, Bruzzone, & Buiatti, 2011; Nolan, Whelan, & Reilly, 2010) were used to identify bad independent components (ICs) that were removed from the data (on average five per subject). Furthermore, the first two epochs of each block and all epochs that followed an incongruent epoch (INC) were removed. Epochs with signal changes exceeding thresholds of 150 μV after ICA artifact removal were excluded, and bad channels that were identified before the ICA were interpolated (only for two subjects, one channel, respectively). For the synchronous condition, on average 11.9% of the congruent epochs and 1.2% of the incongruent epochs were rejected. For the asynchronous condition, on average 12.1% of the congruent epochs and 1.5% of the incongruent epochs were rejected. The relatively high number of congruent epoch rejections results from the rejection criterion that every epoch that followed an incongruent epoch was rejected; it is not due to problems with the data quality of the congruent epochs.

In a final step, grand averages were calculated for the asynchronous (async_CON, async_INC) and the synchronous condition (sync_CON, sync_INC). For these calculations, only standard tone ERPs were considered. The auditory ERPs in response to the deviant tones (congruent and incongruent with the visual information) were not analyzed in the present study. The respective difference waves were calculated for the standard tone conditions, resulting in one difference wave for each stimulus onset asynchrony (SOA) condition: async_INC–async_CON (asynchronous) and sync_INC–sync_CON (synchronous).

The processing of the visual information is of particular importance in the context of our analyses. Therefore, we investigated the number of trials on which a blink occurred around the visual stimulus onset—that is, on which a blink (partly) occluded the visual stimulus presentation. On average, less than 2% of the visual stimuli were partly occluded by a blink (fewer than 4 to 6 trials per condition in half of the subjects). Hence, we are confident that the reported effects in the current study are not influenced by the “blinking behavior” of the participants.

Behavioral data and ERP mean amplitudes within canonical region and time window of interests (ROIs) were tested with Bayesian repeated-measures analyses of variance (ANOVAs) estimating Bayes factors (BF_10_) and with frequentist repeated-measures ANOVAs with identical designs. All statistical analyses were computed in JASP (JASP Team, 2019). For the Bayesian rANOVA, participants’ variation was included as a random factor (*r* = 1), whereby their variance was considered as a nuisance. The calculation of BF_10_ was performed using 50,000 Monte Carlo sampling iterations and a scaling factor *r* = .5 for fixed effects. The null hypothesis corresponded to a standardized effect size δ = 0. We compared all models with the null model (BF_10_). Additionally, BF_Incl_ was calculated across models including a main effect or interaction compared with equivalent models that do not include this effect (see Mathôt, 2017). Data were interpreted as moderate (or strong) evidence in favor of the alternative hypothesis if BF_10_ was ≥3 (or ≥10). If BF_10_ was ≤0.33 (or ≤0.1), this was interpreted as moderate (or strong) evidence in favor of the null hypothesis. Values close to 1 would be only weakly informative and were considered as anecdotal evidence (Lee & Wagenmakers, 2013).

For frequentist repeated-measures ANOVAs, an alpha level of .05 was defined. Statistically significant effects were reported, including the eta squared (ƞ^2^) effect size measure. Significant interactions were investigated by computing follow-up two-tailed *t* tests.

For the behavioral data, the first two trials of each block were removed, and trials that directly followed an incongruent trial were rejected. Furthermore, only responses toward the standard sound (congruent or incongruent with the visual information) were considered for the analyses. A 2 × 2 repeated-measures ANOVA was performed, including the factors SOA (asynchronous vs. synchronous) and congruency (congruent vs. incongruent).

For the ERP mean amplitudes, a 2 × 2 repeated-measures ANOVA was conducted. The ANOVA included the factors SOA (asynchronous vs. synchronous) and congruency (congruent vs. incongruent). The computation of the ERP mean amplitudes was based on canonical region and time window of interests (ROIs), as previously defined by Pieszek et al. (2013) and Stuckenberg et al. (2019). Hence, for the IR, an analysis time window from 105 to 130 ms after sound onset was used. For the calculation of the ERP mean amplitudes, the mean of the electrodes FC5 and C3 was considered for the left hemisphere and the mean of the electrodes FC6 and C4 for the right hemisphere.

In order to confirm the significant elicitation of components in the N2 (185–225 ms) and P3 (235–355 ms) range, the ERP mean amplitudes of the electrodes Fz, FCz, and Cz (frontal ROI midline) were considered, and a 2 × 2 repeated-measures ANOVA with the factors SOA (asynchronous vs. synchronous) and congruency (congruent vs. incongruent) was performed. For the computation of the ERP mean amplitudes in the N2 and P3 range, we chose canonical time windows of interest as previously defined by Pieszek et al. (2013) and Stuckenberg et al. (2019). In contrast to the ROI midline (Fz, Cz, Pz) used in these studies, we chose a frontal ROI midline (Fz, FCz, Cz).

## Results

### Behavioral data

#### RTs

In 97.9 % of trials, a behavioral response was given within the response window. The averages of the response time and accuracy data are displayed in Table 1. The 2 × 2 Bayesian ANOVA favored the model including the SOA and congruency main effects (BF_10_ = 3.823 × 10^16^ ± 0.714%). The data provided strong evidence and significant results for the SOA (BF_Incl_ = 15.091), *F*(1, 18) = 6.335, *p* = .022, ƞ^2^ = 0.035, and congruency main effects (BF_Incl_ = 2.219 × 10^16^), *F*(1, 18) = 157.661, *p* < .001, ƞ^2^ = 0.582. In addition, the data provided anecdotal evidence against the nonsignificant SOA × Congruency interaction (BF_Incl_ = 0.357), *F*(1, 18) = 0.835, *p* = .373, ƞ^2^ = 0.001. Participants’ responses were faster in the congruent than in the incongruent condition. In addition, they responded faster in the asynchronous than in the synchronous condition (see Table 1).

**Table 1:** Mean response times (RTs) in ms and mean accuracy (in %) of behavioral responses for the congruent and incongruent conditions of the asynchronous and the synchronous condition

SOA condition	Congruency condition	RTs (*SD*) in ms	Accuracy (*SD*) in %
Asynchronous	Congruent	251.2 (33.7)	99.9 (0.2)
	Incongruent	369.2 (46.5)	96.7 (3.1)
Synchronous	Congruent	276.3 (31.7)	100 (0.1)
	Incongruent	404.7 (78.9)	93.1 (5.1)

*Note.* Standard deviations (*SD*) are given in parentheses.

#### Accuracy

The corresponding analyses were performed for the accuracy data. The 2 × 2 Bayesian ANOVA favored the model including the SOA and congruency main effects and the SOA × Congruency interaction (BF_10_ = 7.659 × 10^8^ ± 1.628%). The data provided strong evidence for the significant congruency main effect (BF_Incl_ = 5.375 × 10^7^), *F*(1, 18) = 47.291, *p* < .001, ƞ^2^ = 0.387, and moderate evidence for the significant SOA main effect (BF_Incl_ = 4.609), *F*(1, 18) = 7.800, *p* = .012, ƞ^2^ = 0.048, and the significant SOA × Congruency interaction (BF_Incl_ = 7.520), *F*(1, 18) = 8.402, *p* = .010, ƞ^2^ = 0.050. In the follow-up Bayesian *t* test, the data provided strong evidence for the congruency effect in both SOA conditions, which was mirrored by the follow-up two-tailed *t* tests (asynchronous: BF_10_ = 143.179 ± <0.001%), *t*(18) = 4.619, *p* < .001; (synchronous: BF_10_ = 1506.553 ± <0.001%), *t*(18) = 5.852, *p* < .001. Furthermore, response accuracy was similar across SOA conditions for congruent sounds (BF_10_ = 0.310 ± 0.015 %), *t*(18) = −0.777, *p* = .447, but responses to incongruent visual-cue–sound combinations were more accurate when they were presented asynchronously compared with synchronously (BF_10_ = 4.919 ± 0.002%), *t*(18) = 2.847, *p* = .011.

### ERP data

#### IR

**Fig. 2:**
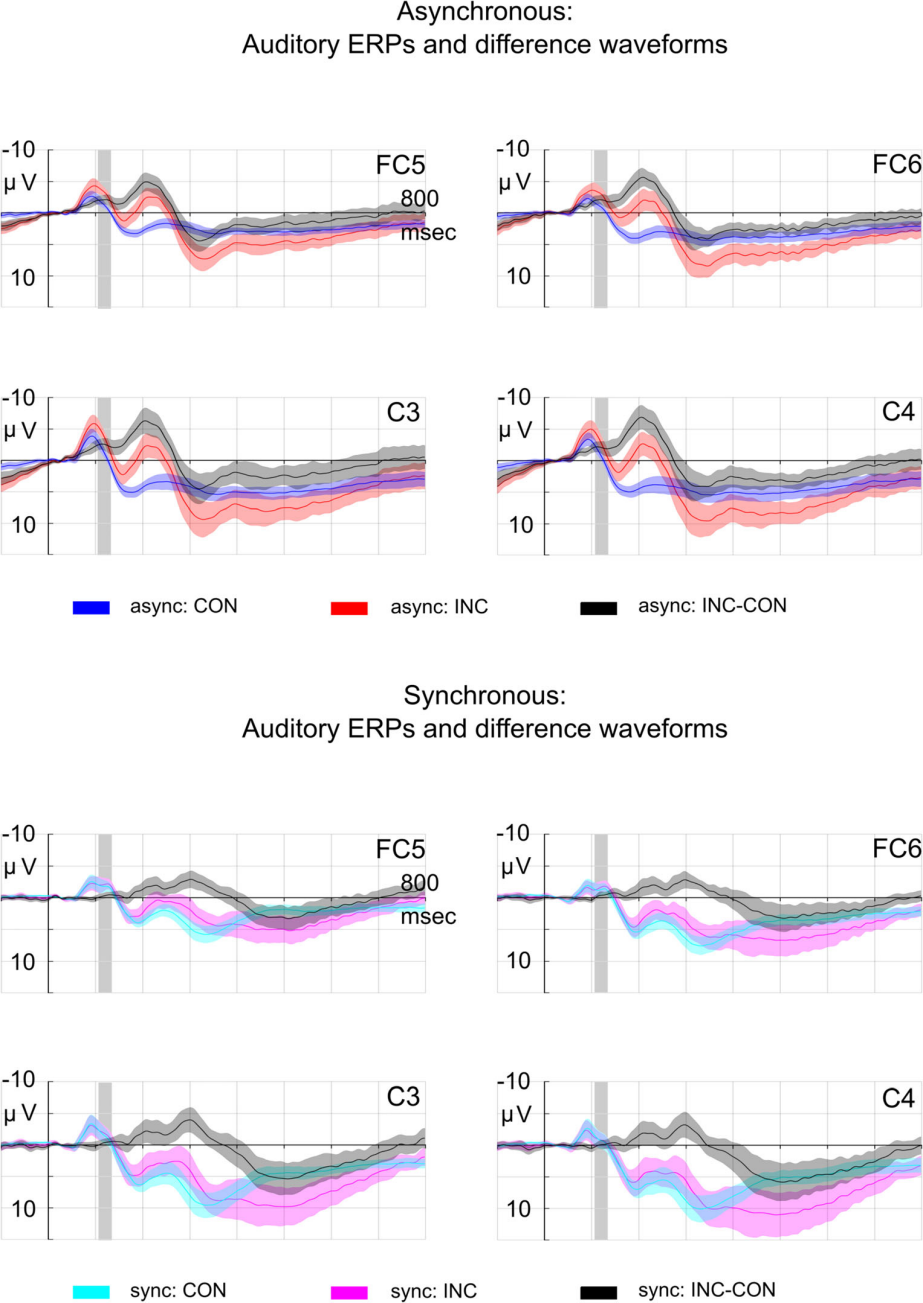
Auditory ERPs and difference waveforms for asynchronous (top) and synchronous (bottom) conditions from −100 to 800 ms relative to sound onset. Grand averages and corresponding 95% confidence intervals (CIs) are displayed. Statistical testing window (105–130 ms) for the investigation of the IR elicitation is indicated with a gray bar. (Color figure online)

An enhancement of the difference wave within the statistical IR time window (105–130 ms) was confined to the asynchronous stimulation condition and not observed in the synchronous presentation condition (see Fig. 2). The 2 × 2 Bayesian ANOVA favored the model including the SOA and congruency main effects and the interaction SOA × Congruency (BF_10_ = 13209.913 ± 2.137%). The data provided strong evidence for the significant congruency main effect (BF_Incl_ = 164.292), *F*(1, 18) = 25.465, *p* < .001, ƞ^2^ = .078, and for the significant SOA × Congruency interaction (BF_Incl_ = 42.464), *F*(1, 18) = 35.084, *p* < .001, ƞ^2^ = .053, but only moderate evidence for the non-significant SOA main effect (BF_Incl_ = 3.071), *F*(1, 18) = 3.915, *p* = .063, ƞ^2^ = .026. In the follow-up Bayesian *t* tests and in the follow-up two-tailed *t* tests, the data provided anecdotal evidence against an effect of congruency (i.e., no difference between congruent and incongruent conditions) in the synchronous condition (BF_10_ = 0.369 ± 0.012%), *t*(18) = 1.001, *p* = 0.330, but strong evidence for an effect of congruency (i.e., significant difference between congruent and incongruent conditions) in the asynchronous condition (BF_10_ = 3054.732 ± <0.001%), *t*(18) = 6.235, *p* < .001.

#### N2b

**Fig. 3.**
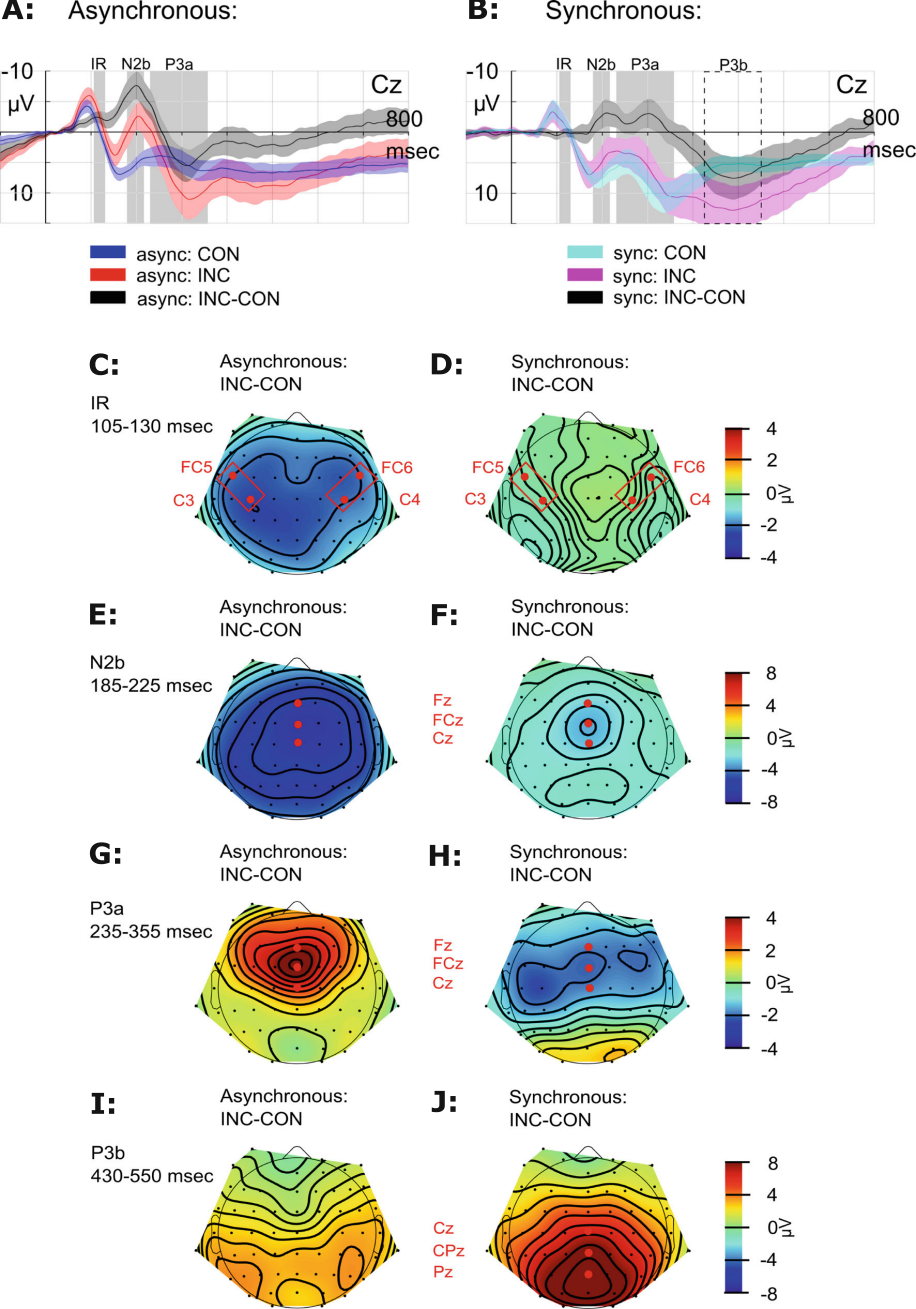
**a**–**b** Auditory ERPs and difference waveforms for asynchronous (left) and synchronous (right) conditions exemplary at the electrode Cz. Grand averages and corresponding 95% confidence intervals (CIs) are displayed. Statistical testing window for the investigation of the IR, N2b and P3a elicitation is indicated with a gray bar. The alternative statistical testing window for the P3b analysis is indicated with dotted lines. Row 2–5: Potential maps (nose referenced) show the scalp distribution within the IR (105–130 ms, **c–d**), N2b (185–225 ms, **e–f**), P3a (235–355 ms, **g–h**), and P3b (430–550 ms, **i–j**) time window of the difference data (incongruent-minus-congruent) for the asynchronous (left) and synchronous (right) conditions. ROIs for statistical analyzes are indicated in red. (Color figure online)

An N2b component was elicited in response to incongruent visual-cue–sound combinations, ERP mean amplitudes in the N2b range (185–225 ms) were more negative in response to incongruent than to congruent sounds (see Fig. 3a–b for an exemplary plot at Cz). The 2 × 2 Bayesian ANOVA favored the model including the main effects of the factors SOA and congruency and the SOA × Congruency interaction (BF_10_ = 1.168 × 10^10^ ± 2.330%). The data provided strong evidence and significant results for all effects (SOA: BF_Incl_ = 9006.793), *F*(1, 18) = 31.843, *p* < .001, ƞ^2^ = 0.156; (congruency: BF_Incl_ = 3.544 × 10^6^), *F*(1, 18) = 37.782, *p* < .001, ƞ^2^ = 0.266; (SOA × Congruency: BF_Incl_ = 18.417), *F*(1, 18) = 21.135, *p* < .001, ƞ^2^ = 0.051. In the follow-up Bayesian *t* tests and in the follow-up two-tailed *t* tests, the data provided strong evidence for an effect of congruency in the asynchronous condition (BF_10_ = 30897.944 ± <0.001%), *t*(18) = 7.553, *p* < .001, and moderate evidence for an effect in the synchronous condition (BF_10_ = 6.568 ± 0.001%), *t*(18) = 3.007, *p* = .008.

One may argue that what we actually observe in the synchronous condition is an IR that is delayed by 100 ms (hence observed ~210 ms after sound onset) and a similarly delayed N2b (around 300 ms; see Fig. 3b). Nevertheless, potential maps show a N2b typical distribution in the time window of 185 to 225 ms (see Fig. 3f) that is distinct from the frontolateral IR distribution that is observed in the asynchronous condition (see Fig. 3c). Hence, we assume that the second negative enhancement that is observed in the difference wave of the synchronous condition around 300 ms reflects most likely a latency difference of P3b elicitation between incongruent and congruent conditions within the synchronous condition rather than shifted IR and N2b components.

#### P3a and P3b

A frontocentrally distributed P3a component was only elicited in response to incongruent visual-cue–sound combinations within the asynchronous condition. In the asynchronous condition, ERP mean amplitudes in the P3a range (235–355 ms) were more positive in response to incongruent visual-cue–sound combinations than to congruent sounds. In the synchronous condition, no P3a component was observed in the canonical time window of interest but a small negativity with a broad frontocentral distribution. The 2 × 2 Bayesian ANOVA favored the model including the main effects of the factors SOA and congruency and the SOA × Congruency interaction (BF_10_ = 7.284 ± 2.255%). The data provided anecdotal evidence against the nonsignificant SOA (BF_Incl_ = 0.294), *F*(1, 18) = 0.432, *p* = .519, ƞ^2^ = 0.004, and moderate evidence against the nonsignificant congruency main effects (BF_Incl_ = 0.400), *F*(1, 18) = 1.739, *p* = .204, ƞ^2^ = 0.009, but strong evidence for the significant SOA × Congruency interaction (BF_Incl_ = 63.517), *F*(1, 18) = 21.052, *p* < .001, ƞ^2^ = 0.086. In the follow-up Bayesian *t* tests and in the follow-up two-tailed *t* tests, the data provided anecdotal evidence for an effect of congruency in the synchronous condition (BF_10_ = 2.061 ± 0.004%), *t*(18) = 2.339, *p* = .031, but strong evidence for an effect of congruency in the asynchronous condition (BF_10_ = 20.829 ± <0.001%), *t*(18) = −3.623, *p* = .002.

In the asynchronous but not in the synchronous condition, a P3a component with a frontocentral maximum of the peak amplitude was elicited (see Fig. 3g–h). In the synchronous condition, we observe a positive enhancement of the incongruent-minus-congruent difference wave (with a parietal maximum of the peak amplitude) at around 500 ms after sound onset (see Fig. 3b, dotted lines, and 3j). In order to investigate the enhancement of the difference wave, we performed a two-tailed *t* test, with the mean of the ERP mean amplitudes at ROI parietal midline electrodes Cz, CPz, Pz elicited within 430–550 ms after sound onset for the incongruent and the congruent conditions within the synchronous condition. We observed strong evidence for an effect of congruency within the synchronous condition (BF_10_ = 543.362 ± <0.001%), *t*(18) = −5.311, *p* < .001. We assume that this results from a delayed and increased P3b in the incongruent trial processing of the synchronous condition compared with an earlier (300–350 ms) and less pronounced elicitation in the congruent condition. In addition, this latency difference presumably causes the second negative enhancement of the difference wave incongruent-minus-congruent around 300 ms after sound onset.

To summarize, we observe a P3a elicitation in the asynchronous condition (235–355 ms after sound onset) and a P3b latency difference between congruent and incongruent ERP mean amplitudes in the synchronous condition (incongruent P3b peak between 430 and 550 ms after sound onset).

## Discussion

### IR elicitation

We measured ERPs in response to auditory stimuli that either matched (congruent) or conflicted with (incongruent) visual stimuli. We compared the auditory processing of a condition where a visual-based prediction can be established (asynchronous presentation of preceding visual and succeeding auditory stimuli) and a condition where no visual-based prediction can be established since the visual and auditory stimuli are presented simultaneously (synchronous condition). Only in the asynchronous condition, incongruent compared with congruent but otherwise identical sounds elicited enhanced negativity around 105–130 ms after auditory onset (IR). The auditory system integrates preceding visual symbolic information on a sensory level in order to predict upcoming auditory events. In the synchronous condition, the integration of the visual information apparently takes place later in time and at higher cognitive levels.

The elicitation of the IR in the asynchronous condition as well as the topographical distribution (frontolateral distribution; see Fig. 3c), behavioral, N2b, and P3a processing replicate the findings of Pieszek et al. (2013), Stuckenberg et al. (2019) and Widmann et al. (2004). In accordance with their findings, we suppose that the IR reflects sensorial prediction error rather than bimodal feature mismatch between fast extracted auditory and visual information. In line with the predictive coding theory (Friston, 2012; Friston & Kiebel, 2009; Garrido et al., 2008), it is assumed that the auditory representation is “preactivated” by the preceding visual information. More precisely, the neurons that are responsive to the sound are preactivated by the preceding visual information via feedback connections in the predictive layer. In other words, top-down information is used to preactivate the corresponding neurons. Hence, the prediction error signal is reduced if the expected sound (congruent sound) is presented compared with when an unexpected sound (incongruent sound) is presented.

In the synchronous condition, the simultaneously presented visual information does not have the capability to preactivate the auditory representation from a top-down level. Nevertheless, it could have been that the regularly presented visual-cue–sound combination formed a bimodal percept. The violation of this bimodal percept (in our case by the differing visual input) could have resulted in a bimodal feature mismatch signal. Our findings suggest that within the synchronous condition, no such mechanism was active, since we do not observe an enhanced response for incongruent compared with congruent sounds within the IR time window. This leads to the assumption that the visual information was not integrated in the auditory object representation in the sense of a bimodal feature representation, at least not on a sensory level.

Based on the findings of Ullsperger et al. (2006)—MMN modulation by visual information—we would have expected an IR elicitation in the synchronous condition. Ullsperger et al. (2006) presented a picture of a hammer hitting a nail simultaneously with a “bang” sound as the standard visual and auditory presentation (79% of trials). They introduced a condition—similar to our incongruent condition—where the deviant picture was presented together with the standard sound (9.9% of trials). They observed a negative deflection around 100 ms after sound onset of the difference wave between ERPs elicited by a standard sound presented with a deviant picture (incongruent condition) and ERPs elicited by a standard sound presented with a standard picture (congruent condition). We were not able to replicate these findings within our synchronous condition even though the experimental setup and the presentation probability of visual-cue–sound combinations was similar to Ullsperger et al. (2006). Although Ullsperger et al. (2006) report a simultaneous visual and auditory stimulus presentation, they illustrate in the description of the experimental setup that the pictures actually were presented 100 ms “with a smooth fading in onset” before the onset of the sound. While the actual parameters of the smooth onset remain unclear, the slight asynchrony might be already sufficient to establish a visual based prediction. In this case, the results would be in line with our findings, but to confirm that 100-ms precedence of the visual information are sufficient to establish a visual based prediction, an empirical validation would be required.

To summarize, we showed that precedence of visual information is necessary to modulate early auditory processing (i.e., to generate visual-based predictions). This is in line with the studies contrasting bimodal and unimodal information, showing that preceding visual information is necessary to modulate N1. For instance, Vroomen and Stekelenburg (2010) indicate that only preceding visual information leads to an N1 suppression effect that possibly reflects expectation of the sound (i.e., audio-visual integration, as they term it).

In conclusion, the IR most likely reflects a sensory prediction effect, thus, a preactivation of the expected auditory event based on the preceding visual information. Our findings do not exclude the interaction of visual and auditory information at a sensory level per se. We only show that, for symbolic information in a trial-by-trial paradigm, visual information can only modulate sensory auditory processing if it precedes the auditory stimulus.

### Subsequent processing

In the asynchronous and synchronous condition, a significant N2 component was elicited. The N2 component is usually used as a marker of early phase auditory change detection (e.g., in the oddball paradigm: Näätänen, Simpson, & Loveless, 1982; Novak, Ritter, Vaughan, & Wiznitzer, 1990; Patel & Azzam, 2005). We observe an N2 component with anterior scalp distribution (see Fig. 3e–f). This type of N2 component can be divided into separate control-related and mismatch-related subcomponents (Folstein & Van Petten, 2008). Hence, we presumably observe a deviance-related or novelty N2b.

Lindström, Paavilainen, Kujala, and Tervaniemi (2012) investigated the effects of temporal aspects and cognitive demands during audiovisual deviance-detection tasks on the N2 elicitation. They presented three tone categories (low, middle, or high tones, five per category) and three visual categories (opal-shaped white circles, either large, medium, or small) either simultaneously or with a visual precedence of 300 ms. They observed that N2 peaked (at electrode Cz) at about 250 ms in the asynchronous condition and at about 500 ms in the synchronous condition. The N2 amplitude was largest when the visual information was preceding the auditory stimulus and a simple bimodal association rule was used. They argue that the underlying mechanisms are probably not different since the topographies indicate no difference. Lindström et al. (2012) suggest that the amount of visual precedence (relative to auditory onset) and the nature of the stimulus materials together modulate the processing of visually induced auditory expectations.

These findings are in line with our observations. In the synchronous condition, the N2b component is slightly delayed and not as pronounced as in the asynchronous condition, but the topographies indicate no difference between the two conditions (see Fig. 3e–f).

Furthermore, in the asynchronous, but not in the synchronous condition, a P3a component was elicited between 235 and 355 ms after sound onset. The potential maps (see Fig. 3g) reveal a frontocentral maximum of the peak amplitude. This characteristic as well as the relatively short peak latency lets us assume that we actually observe a P3a component that has been observed to be elicited in response to infrequent distinct tones or distractors (Polich, 2007).

In the synchronous condition, no P3a was elicited. But the analysis of the additional time window (430 to 550 ms after sound onset) revealed a later starting P3b component in the incongruent condition. The potential maps (see Fig. 3j) reveal a central-parietal maximum of the peak amplitude speaking for a P3b elicitation. The P3b component has been associated with context updating operations and subsequent memory storage (Polich, 2007). In the synchronous condition, the incongruency of visual and auditory information is processed, but in contrast to the asynchronous condition, no prior expectation was made, since the visual information was not preceding the auditory information. In other words, in the synchronous condition, the actual bimodal mismatch between the visual and auditory information is encountered and evaluated, but independent of a visual-based prediction. In addition, there is a latency difference in the synchronous condition between the P3b elicitation in the incongruent and congruent condition, which results into the second negative enhancement of the difference wave at around 300 ms (see Fig. 3b).

Moreover, stimulus evaluation time is prolonged in the synchronous condition (as observed in the behavioral results). Since P3 latency is thought to be proportional to stimulus evaluation timing (Kutas, McCarthy, & Donchin, 1977; Polich, 2007), the P3 latency difference between the asynchronous and synchronous condition could be explained by the differences in stimulus evaluation. In addition, more recent studies found longer P3 latencies for deviant processing when visual and auditory information are presented simultaneously (Andres, Oram Cardy, & Joanisse, 2011). To conclude, we most likely observe a P3a elicitation in the asynchronous condition and a P3b latency difference between incongruent and congruent ERP mean amplitudes in the synchronous condition.

To summarize, we observe N2b and P3 (asynchronous: P3a; synchronous: P3b) elicitation in both SOA conditions, which presumably also reflects deviant detection (i.e., prediction error-related processes). This contrasts with the finding that the IR is confined to the asynchronous condition. In brief, the ability to detect an incongruent visual-cue–sound combination at higher hierarchical levels is probably independent of whether they are presented asynchronously or synchronously.

### Behavioral measures

We observe a significant elicitation of the congruency effect in both asynchronous and synchronous presentation conditions. Participants respond faster and more accurately with congruent than with incongruent stimulation. Interestingly, participants responded faster in the asynchronous condition than in the synchronous condition, whereby the accuracy of their responses only differed in response to incongruent trials (responded more accurate in the asynchronous than in the synchronous condition). These findings are in line with the observations of Lindström et al. (2012). They showed that incongruency was more easily detected in an asynchronous (300 ms preceding) than in a synchronous (no-delay) condition. Response times, similar to our study, were faster in the asynchronous than in the synchronous condition.

In contrast to the ERP processing—revealing delayed processing in the synchronous compared with the asynchronous condition—the behavioral congruency effects are relatively similar in the asynchronous and the synchronous condition. The mean difference between incongruent and congruent response times in the asynchronous condition is 117.96 ms, and in the synchronous condition is 128.42 ms. On the one hand, there is a significant difference between conditions, but on the other hand, the difference is not as pronounced as we would have expected based on the differences observed in the ERPs. Only small and relatively late effects of audiovisual (in-)congruency were observed in the synchronous presentation condition, while the observed behavioral effects were actually larger. To conclude, the behavioral results reveal a beneficial effect of the visual preceding information on response time and response accuracy.

## Conclusions

Based on the existing literature, we assumed that tones with their pitch being incongruent with the location of the visual stimulus elicit the IR. It was only observed in the condition where the visual information preceded the auditory information (asynchronous condition) and not when they were presented simultaneously (synchronous condition). Within the asynchronous condition, we replicated the findings of Pieszek et al. (2013), Stuckenberg et al. (2019) and Widmann et al. (2004) regarding IR elicitation and topography, N2b and P3a elicitation as well as behavioral results. Within the synchronous condition, we observed incongruency effects only at N2b and P3a levels. These findings support the sensorial prediction error hypothesis stating that the amplitude of auditory evoked potential around 100 ms after sound onset is enhanced in response to unexpected compared with expected, but otherwise identical sounds. It suggests that the human auditory system establishes sensory representations of expected auditory events based on predictive and preceding visual information whereby bimodal feature mismatch is processed at higher cognitive levels, at least in a trial-by-trial setup.

## Supplementary Information

ESM 1 (DOCX 145 kb)
