# Supplementary material for: Modulation of early auditory processing by visual information: Prediction or bimodal integration?
Source: Atten Percept Psychophys. 2021 Jan 27;83(4):1538–51. doi: 10.3758/s13414-021-02240-1 (PMC8084811; doi:10.3758/s13414-021-02240-1)
Supplement: Supplementary file 1 — (DOCX 145 kb) [file 13414_2021_2240_MOESM1_ESM.docx]

**Supplementary Material**

**Asynchronous condition:**

difference wave (INC – CON) with different filter settings


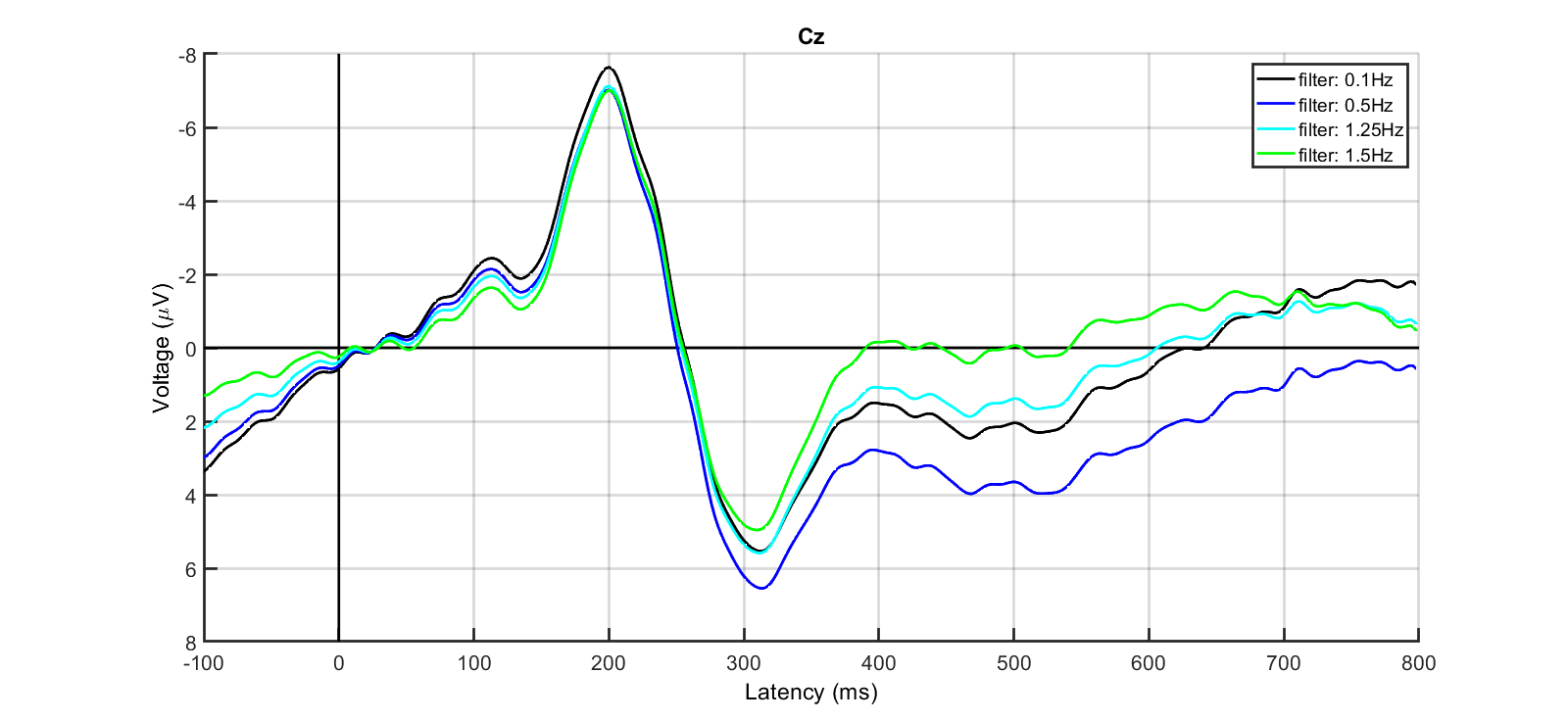


**Synchronous condition:**

difference wave (INC – CON) with different filter settings


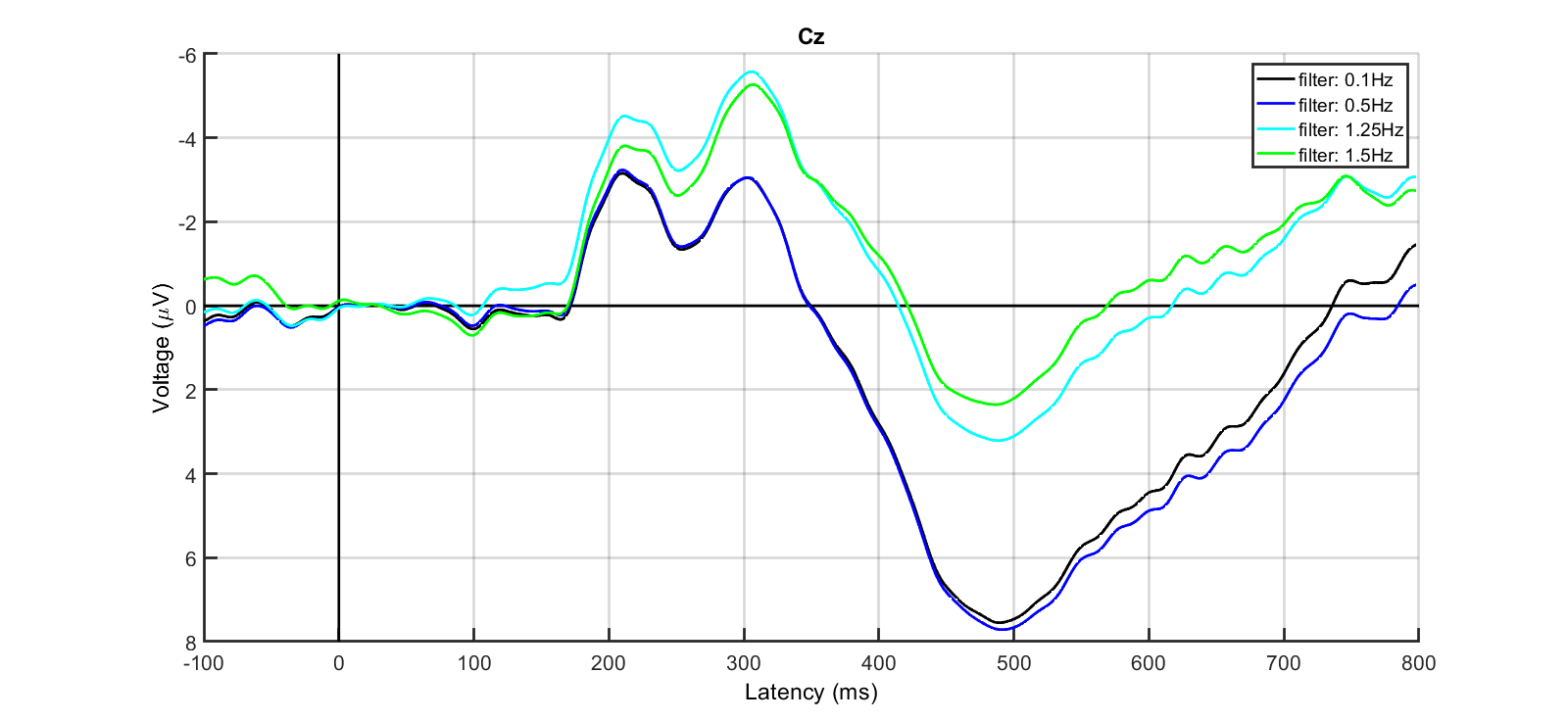


**Figure S1:** Difference waveforms (INC – CON) for asynchronous (**top**) and synchronous (**bottom**) conditions from -100 to 800 ms relative to sound onset. Different high-pass filter settings are contrasted (**black:** 0.1 Hz high-pass filter cutoff, **blue:** 0.5 Hz high-pass filter cutoff, **cyan:** 1.25 Hz high-pass filter cutoff, **green:** 1.5 Hz high-pass filter cutoff). The data was low-pass filtered with 48 Hz cutoff and baseline corrected from 0 to +50 ms.
